# Highly Sensitive Humidity Sensor Based on Oblique Carbon Nanoplumes

**DOI:** 10.3390/s18103407

**Published:** 2018-10-11

**Authors:** Siqi Qiao, Xiaoyan Peng, Lidan Wang, Shukai Duan, Jin Chu, Pengfei Jia

**Affiliations:** 1 Chongqing Key Laboratory of Non-Linear Circuit and Intelligent Information Processing, College of Electronic and Information Engineering, Southwest University, Chongqing 400715, China; qiao47@email.swu.edu.cn (S.Q.); ldwang@swu.edu.cn (L.W.); duansk@swu.edu.cn (S.D.); jiapengfei@swu.edu.cn (P.J.); 2 Chongqing Key Laboratory of Multi-Scale Manufacturing Technology, Chongqing Institute of Green and Intelligent Technology, Chinese Academy of Sciences, Chongqing 400714, China; chujin@cigit.ac.cn

**Keywords:** carbon nanomaterial, hot filament physical vapor deposition, humidity sensor

## Abstract

In this work, we fabricated three carbon nanoplume structured samples under different temperatures using a simple hot filament physical vapor deposition (HFPVD) process, and investigated the role of surface morphology, defects, and graphitic content on relative humidity (RH) sensing performances. The Van der Drift growth model and oblique angle deposition (OAD) technique of growing a large area of uniformly aligned and inclined oblique arrays of carbon nanoplumes (CNPs) on a catalyst-free silicon substrate was demonstrated. The optimal growing temperature of 800 °C was suitable for the formation of nanoplumes with larger surface area, more defect sites, and less graphitic content, compared to the other samples that were prepared. As expected, a low detection limit, high response, capability of reversible behavior, and rapid response/recovery speed with respect to RH variation, was achieved without additional surface modification or chemical functionalization. The holes’ depletion has been described as a RH sensing mechanism that leads to the increase of the conduction of the CNPs with increasing RH levels.

## 1. Introduction

The detection and control of humidity are important considerations for different applications including food quality monitoring, meteorology and medical equipment functioning. Recently, carbon nanomaterials have been investigated as a promising candidate for sensing applications due to their comparatively high conductivity, high mechanical stiffness, large sensing surface area, and high chemical inertness [1,2,3]. Specifically, the strong interaction with adsorbed water molecules makes carbon nanomaterial suitable candidates for building relative humidity (RH) sensors [4].

The morphological features and structure include: surface area, graphitic contents [5], number of conducting electrons [6], defects within nanostructures [7], and surface functionalities [8] which all play important roles in the carbon-based sensor response. Many efforts, including controlling structure, doping, and optical excitation, have been made to improve sensor performances. For example, the oblique angle deposition (OAD) technique is regarded as an effective method to generate porous one-dimensional nanostructures, a result of the self-shadowing nature of the deposition process [9], which possesses an extremely high surface area and is an important factor for gas sensing applications.

Usually carbon adsorbs numerous oxygen derivations on the surface, which makes the carbon surface hydrophilic, and enhances water vapor adsorption [10]. Upon adsorption of water molecules on the carbon’s surface, electrons transfer between the carbon and water molecules. The transfer of the electrons leads to the holes’ depletion or enrichment of p-type semiconductors and consequently decreases electrical conductivity. On the other hand, it was also reported that water vapor is chemisorbed on the surface of the semiconductor sensing materials, and consequently leads to the formation of the hydroxyl group on the surface [11] and proton transfer occurs following Grotthuss’s chain reaction [10].

The originality of this work involves providing a comparison of the sensor response of carbon nanostructures synthesized at different temperatures with a simple OAD technique by hot filament physical vapor deposition (HFPVD). Dinh et al. [12] recently reported an inexpensive, environmentally-friendly and flexible thermal flow sensor by using pencil graphite. In this work we have demonstrated a simple approach to grow carbon nanoplumes by using graphite stick as a precursor. Scanning electron microscopy (SEM), transmission electron diffraction (TED), and Raman spectroscopy measurements reveal that the preparation temperature has a moderate effect on morphology and structure of the carbon samples. Thereafter, the sensitivity, reproducibility, response and recovery speed, and transient behavior of the prepared carbon nanostructures towards RH has been examined by measuring variations in the conductivity of the sensing layers, followed by a RH sensing mechanism analysis of the carbon nanoplumes (CNPs). The results indicate that the carbon nanostructures demonstrate excellent performance as RH sensors under different RH ranges.

## 2. Experiment Details

### 2.1. Preparations of the Carbon Nanostructures

Carbon nanostructures were synthesized on 1 cm × 1 cm Si substrates in a HFPVD chamber. A similar fabrication process has been described in our previous publication [13]. In order to prepare a carbon-based sensor with optimum performance, we proceeded to perform a series of depositions under different temperatures. The graphite sticks (0.5 mm in diameter and 15 mm in length) were employed as a carbon source to provide sufficient carbon vapor, and as well, to act as filaments, so that no extra filaments were needed. The Si substrates were firstly ultrasonically washed in a methanol solution for 5 min, rinsed with acetone, and dried with helium. After placing the substrate in a holder, beneath the graphite stick with a distance of 1 cm in the HFPVD chamber, the pressure of the chamber was pressurized down to 10^−3^ mTorr, and then Ar gas was fed into the chamber until the ambient pressure was attained, followed by pressurizing down to 10^−3^ mTorr again. This process was repeated three times in order to keep air out and obtain as good a vacuum as possible. A direct current (DC) power supply was used to heat the filament to promote the carbon gas phase activation. For all the experiments, the incident angle of the plasma deposition was controlled at approximately 45° and the deposition duration was kept at 20 min. The sputtered Cr/Au double-layer electrodes were grown on both sides of the carbon films by using shadow masks, after which the carbon samples were transferred to a quartz tube furnace to anneal at 1000 °C for one hour in a nitrogen atmosphere and then slowly cooled down to 25 °C at a rate of 1.7 °C/min in order to reduce the stress and strain in the films as much as possible. The three CNPs samples prepared by this process at 600, 700 and 800 °C will henceforth be referred to as CNPs-600, CNPs-700, and CNPs-800, respectively.

### 2.2. Characterizations

The morphologic surface and the cross section of the samples obtained were investigated by SEM and TED. Room temperature (RT) Raman spectroscopies were measured by the Jobin-Yvon T64000 Triple-mate system with the coherent argon ion laser emitting radiation of 514.5 nm. A liquid nitrogen (LN_2_) cooled charge-coupled device was used to collect and process the scattered data (purchased from the HORIBA Group, Kyoto, Japan).

### 2.3. Measurement of Sensing Properties

After characterizations, the Au electrodes were sputtered on both sides of the three samples to fabricate the sensor prototypes. The sensing measurement setup used in this work has been described in detail elsewhere [14]. The sensor prototypes were placed in a small stainless steel chamber with a capacity of 1 L, so that the variation of RH level was instantaneous, which is crucial for the accurate measurements of the transient behavior and the response/recovery speed of the sensors. Several gas lines and valves were used to transfer gases and control RH levels inside the test chamber, and a dryer device was installed to dry the sensors. The sensing material inside the chamber was serially connected with an external precise resistor (R = 1000 Ω), which is easy to connect to an external alternating current (AC) power supply (1 V, 1000 Hz) to form a voltage-current-resistor (V-I-R) electrical circuit. The utility of the AC supply avoids the polarization effect which frequently occurs under a direct current (DC) circuit due to the effect of physisorption of water [15,16].

Upon exposure to different RH levels, the sensing material will vary its impedance, which will be sensed as a variation of voltage drop across the precise resistor. The variation of the sensors’ impedance was calculated based on the real-time detection of the voltage drop across the precise resistor according to the formula:

(1) $$Z_{sensor}/Z_{precise}=V_{sensor}/V_{precise}$$

where the $Z_{sensor}$, $Z_{precise}$, $V_{sensor}$ and $V_{precise}$ are the impedance and voltage drop across the carbon based sensors, and the precise resistor, respectively.

The sensors were exposed to dry air flow at 500 sccm at RT for 20 min to record the initial sensor impedance, and in preparation for monitoring the impedance change upon exposure to water vapor. Then the required humidity level was obtained by mixing different percentages of humid air and dry air, and waiting for the impedance of the sensors to stabilize. The temperature of the sensors was detected and controlled by a thermocouple and heater.

## 3. Results and Discussion

**Figure 1:**
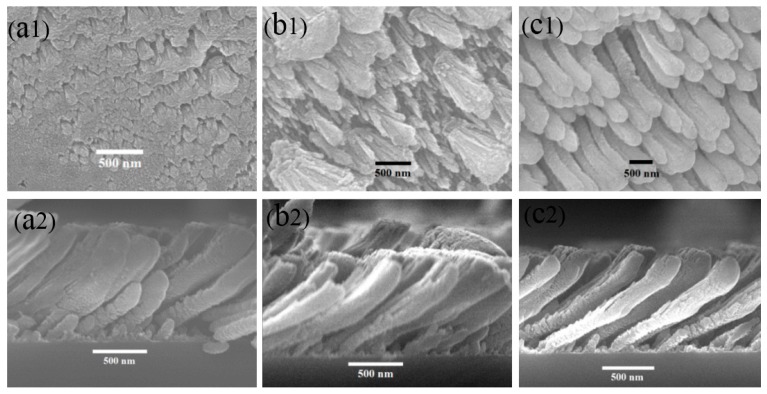
SEM and the corresponding cross-sectional images of (**a1**,**a2**) CNPs-600, (**b1**,**b2**) CNPs-700, and (**c1**,**c2**) CNPs-800.

Figure 1 shows the typical SEM images and the corresponding cross-sectional images of the synthesized carbon samples at temperatures of 600 °C, 700 °C, and 800 °C, respectively. Various nanostructures with different sizes can be easily observed for the carbon grown at different temperatures within otherwise identical growth conditions. When the temperature was 600 °C, nanoplumes were formed on the surface of the Si substrate. When the temperature was increased to 700 °C, nanoplumes have been grown with a length of 1 μm and diameter of 100 nm initially, and gradually increasing to 300 nm for the end part. By further raising the substrate temperature to 800 °C, the surface of the nanoplumes becomes rough and porous. Furthermore, the diameter of the nanoplumes decreased to approximately 200 nm. The corresponding cross-sectional images exhibit oblique nanoplumes with the same angle of approximately 45° to the normal of the three substrates, as shown in Figure 1(a2,b2,c2), however, the length-to-diameter ratio of the nanoplumes increased with the temperature.

Since the carbon plasma was deposited on substrates with an oblique incident angle, the orientation nucleus was randomly developed on the substrate and yielded a geometric shadow region at the initial stage of the carbon vapor deposition [17]. Subsequently, flux prefers to grow on the top of the nuclei, which induces larger values in the height of the nucleus. The Van der Drift model gives rise to the formation of columnar structures. The size of the carbon nuclei and plasma density were varied with the deposition rate determined by deposition temperature [18,19], and consequently this results in different length-to-diameter ratios and surface morphologies.

**Figure 2:**
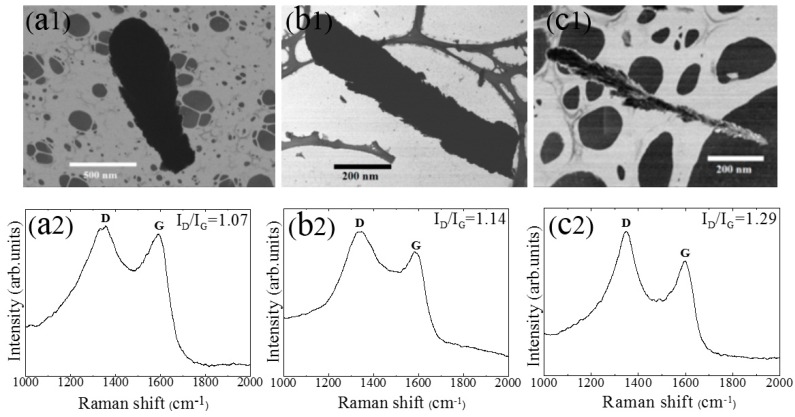
TED BF images and Raman spectra of (**a1**,**a2**) CNPs-600, (**b1**,**b2**) CNPs-700, and (**c1**,**c2**) CNPs-800.

Figure 2(a1,b1,c1) shows the TED bright field (BF) images of the CNPs. The obvious rough surface and porous structure of the CNPs-800 in Figure 2(c1) are indicative of an extremely high surface area. The vaporizable impurities from the residue gas or contamination at high temperatures during deposition may induce a coarse and porous surface on the nanostructure [14]. Two characteristic graphite peaks are observed in the Raman spectra in Figure 2(a2,b2,c2). The pronounced D band at 1352 cm^−1^ is characteristic of high defects in the structure, and the G band at 1582 cm^−1^ is associated with organized graphitic structures of carbon materials [19,20]. Increasing the preparation temperature from 600 °C to 800 °C increased I_D_/I_G_ from 1.07 to 1.29, showing that the defects increase slightly with increased preparation temperature. The high impurity vapor induced at high temperatures might explain the main reason for the increase in defects [14], which obviously can provide more adsorption sites for oxygen molecules on the surface. The CNPs-800 shows stronger and sharper peaks (D and G) in the Raman spectrum than CNPs-600 and CNPs-700 as shown in Figure 2(a2,b2,c2), indicating better quality of the crystalline structure [20,21,22], from which we might conclude that increasing deposition temperature improves the crystalline quality of the carbon nanoplumes in this deposition system.

**Figure 3:**
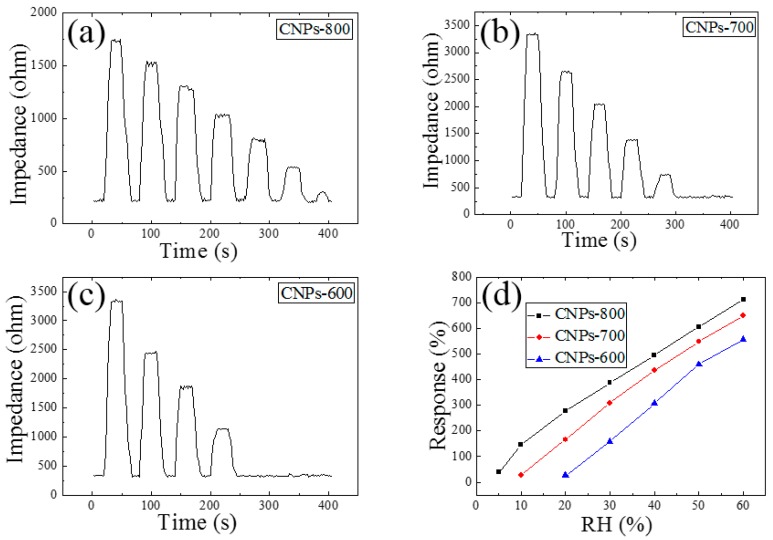
Transient response behavior of (**a**) CNPs-600, (**b**) CNPs-700, (**c**) CNPs-800, and (**d**) the response comparison of three sensors to RH at 200 °C.

The resistive response is defined as $\Delta Z/Z_{0}=\left(Z-Z_{0}\right)/Z_{0}$, where $Z_{0}$ is the initial impedance of the sensor and Z is the impedance after exposure to water vapor. The initial impedances of the three CNPs are varied under different RH levels, which may be principally caused by the surface morphology and defect sites in the samples. Figure 3a–c depicts the transient response behavior of the three CNPs based sensors with decreasing RH at 200 °C. The CNPs-600 and CNPs-700 based sensor were not able to detect the RH below 30% and 20%, respectively, while 5% RH was clearly detectable by the CNPs-800 based sensor with low noise level. The higher graphitic content of CNPs-600 and CNPs-700 relative to CNPs-800 (as illustrated in the Raman spectra) likely restricted access of gas molecules into the innermost portion of the carbon [23] and as a result, low detection limits were not achieved. Figure 3d depicts the impedance-RH behavior of three carbon nanostructure-based sensors at 200 °C. The sensitivity of the three samples decreases linearly with almost similar steps in the range from 60% to 5% RH and the CNPs-800 has the highest sensitivity to water vapor at every RH level. Henceforth we focus on the sensing performance of the CNPs-800 based sensor with the following sensor characterizations.

**Figure 4:**
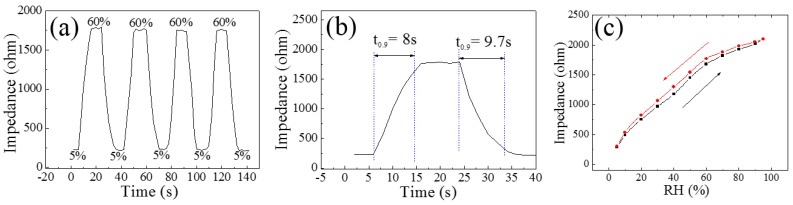
(**a**) Transient response behavior; (**b**) response and recovery time, and (**c**) absorption-desorption behavior of the CNPs-800 based sensor at 200 °C.

The transient response behavior of the CNPs-800 based sensor to dynamic switches of the RH levels between 5% and 60% at 200 °C is exhibited in Figure 4a. When the RH level was switched from 5% to 60%, the impedance of the CNPs-800 rapidly increased to a stable value. When the humidity level was again reduced back to 5% RH, the impedance rapidly decreased to a relatively stable value. This process has been repeated for more than three cycles, which indicates the CNPs-800 based sensor was able to recover the signal after a sensing event, making it capable of repeatable or reversible sensing with unimpaired signals. It is noteworthy that it only takes 8 s for the sensor to increase humidification from 5% to 60% RH, while the recovery time for dehumidification from 60% to 5% RH is about 9.7 s, as shown in Figure 4b. Furthermore, the response can be calculated to be as high as 703% without the aid of UV light or heat treatment during the sensing measurement process. This is different from other carbon-based RH sensors, which usually have high irreversibility and therefore plasma treatment [24], or high testing alternating current frequencies [25] are needed. Figure 4c displays the humidity hysteresis characteristic of the CNPs-800 based sensor. The RH level begins from the detection limit of CNPs-800 (5% RH) and increases to 95% RH, and is then dehumidified back to 5% RH. The maximum humidity hysteresis of the sensor is about 7.40% with a 40% RH. Interestingly, the increasing speed of the impedance becomes slower, compared to the increasing rate at lower impedance when the RH is higher than 60%.

The sensitivity (*S*) of the sensor to RH is defined as:

(2) $$S=\frac{\Delta Z/Z_{0}}{\Delta (\%\mathrm{RH})}$$

The comparison of sensing performance, including sensitivity and response/recovery time, between our work and previous reports about carbon nanomaterial based humidity sensors [16,25,26,27,28,29,30,31], are exhibited in Table 1. Some of these works obtained fast response or recovery behaviors [15,26,27,30]. However, the sensitivity of the CNPs in our work is higher than previous works, which may be due to the large surface area, high defects and low graphitic content of the CNPs-800 based sensor in this work.

**Figure 5:**
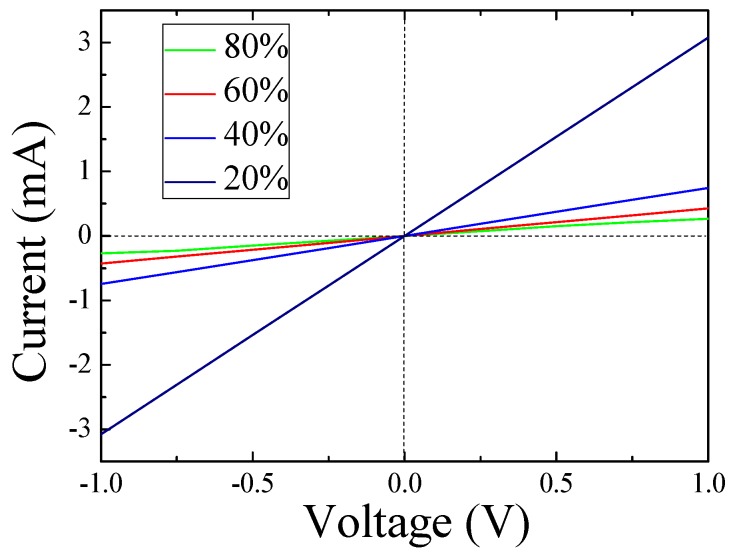
The I–V characteristics of the CNP-800 under different humidity level at RT.

Figure 5 exhibits the typical I–V curves of the CNP-800 based sensor for different static air RH between 20–80% at RT. The vertical and horizontal axes denote the current sent through the sample and the voltage drop across the sample, respectively. The observed I–V curves show strictly linear behavior, which indicates that good ohmic contact between the CNP-800 based sensor and the gold electrodes was obtained.

**Figure 6:**
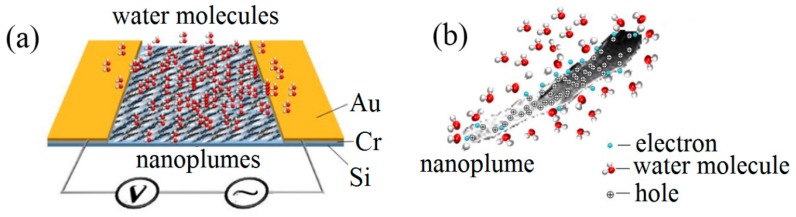
The illustration of (**a**) CNPs-800 at low humid ambient, and (**b**) charge transport between water molecules and one nanoplume.

The carbon nanostructures are normally hydrophilic due to the numerous adsorbed oxygen derivatives [10]. Consequently, water vapor molecules tend to be adsorbed on the surface [10], as exhibited in Figure 6a. The adsorbed water molecules donate electrons to the valence band of the CNP-800, and consequently, this leads to the holes’ depletion (illustrated in Figure 6b). Hence, reduction of conductivity will occur as a result of the increasing separation between the Fermi level and the valence band [8,32]. The thick water vapor layers were physisorbed with an increase in RH, which provides more electrons to the CNPs and further increases the impedance. However, the increase speed became slight slower at higher RH levels, which may be due to the different electron transport mechanisms between the sensitive surface of the CNPs sample and the thicker water vapor layers.

## 4. Conclusions

In conclusion, three CNPs samples have been obliquely deposited using the HFPVD techniqueunder different temperatures. The morphologies, defect sites and graphitic content, develop with temperature, which has been exhibited by SEM and TED images, and Raman spectra. The Van der Drift model and OAD technique has been attributed to the growth mechanism of the CNPs. The optimum temperature of growing nanoplumes with large surface area, high defect and low graphitic content was 800 °C, which induces best sensing performances by comparing the sensing characteristics among three CNPs samples.

The detection limit of the CNPs-800 based sensor was investigated with a RH as low as 5%, and the response of the CNPs-800 was calculated as 703% at 60% RH. The transient response behavior indicates high response, good reproducibility, rapid response and recovery speed of the sensor to RH. Furthermore, for the small hysteresis characteristic, a maximum value of 7.40% was obtained. The holes’ depletion was caused by the electron donation released by the adsorbed water molecules and consequently increased the impedance of the CNPs-800 samples. The comparison of the sensing performance between the CNPs-800 based sensor and other carbon nanomaterial-based humidity sensors indicates that the CNPs samples synthesized by the HFPVD process are a promising candidate for RH sensing applications.

## Figures and Tables

**Table 1:** The comparison of the sensing performances between our work and previous results.

Materials	Sensitivity	Response Time	Recovery Time	Ref.
PI ^a^/MWCNT (2.0 wt %)	0.0018 (20–90% RH)	5 s	-	[16]
MWCNTs	0.0028(11–75.5% RH)	16 s	8 s	[25]
MWCNT network	0.0056 (25–95% RH)	3 s	25 s	[26]
PEI ^b^/MWCNT (Layer-by-layer)	0.0099 (5–85% RH)	2 s	30 s	[27]
a-C ^c^ film/n-Si	0.0238 (11–95% RH)	~3 min	~4 min	[28]
KC ^d^-MWCNT-G ^e^	0.025 (20–80% RH)	50 s	100 s	[29]
BCB ^f^, 4024-40, Dow Chemical	0.0250 (50–90% RH)	0.5 s	4.5 s	[30]
Carbon Nanosheets	0.033 (11–40% RH)	30 s	90 s	[31]
Carbon Nanoplumes	0.117 (5–65% RH)	8 s	9.7 s	ours

^a^ Polyimide; ^b^ Polymethylmethacrylate; ^c^ Amorphous Carbon; ^d^ Kappa-Carrageenan; ^e^ Glycerin; ^f^ Benzocyclobutene.
